# Pigeons and the Ambiguous-Cue Problem: A Riddle that Remains Unsolved

**DOI:** 10.3389/fpsyg.2017.00941

**Published:** 2017-06-08

**Authors:** Óscar García-Leal, Carlos Esparza, Laurent Ávila Chauvet, Héctor O. Camarena-Pérez, Zirahuén Vílchez

**Affiliations:** Center for Studies and Investigations in Behavior, University of GuadalajaraGuadalajara, Mexico

**Keywords:** ambiguous-cue problem, interfering cue hypothesis, value transfer theory, pigeons, partial reinforcement

## Abstract

The ambiguous-cue task is composed of two-choice simultaneous discriminations involving three stimuli: positive (P), ambiguous (A), and negative (N). Two different trial types are presented: PA and NA. The ambiguous cue (A) served as an S- in PA trials, but as an S+ in NA trials. When using this procedure, it is typical to observe a less accurate performance in PA trials than in NA trials. This is called the ambiguous-cue effect. Recently, it was reported in starlings that the ambiguous-cue effect decreases when the stimuli are presented on an angled (120°) panel. The hypothesis is that the angled panel facilitates that the two cues from each discrimination are perceived as a compound, precluding value transfer via a second-order conditioning mechanism. In this experiment, we used pigeons and a flat panel. Nevertheless, our data were quite similar to the previous data in starlings. We conclude that the form of the panel cannot explain the ambiguous-cue effect. Several alternatives to be explored in future experiments are suggested. The riddle of the ambiguous-cue problem still remains unsolved.

## Introduction

The ambiguous-cue problem is a well-documented phenomenon in literature and is relatively simple. It is typically observed in two simultaneous binary discriminations employing three stimuli. One of these stimuli (P) is always reinforced, another (N) is never reinforced, but the third (A), is reinforced in the presence of N, but never reinforced in the presence of P. In this context, A is the ambiguous-cue because it will either be reinforced or not depending on the stimulus with which it is associated. Discriminations are usually denoted as PA trials and NA trials (Vasconcelos and Monteiro, 2014). The result is a less accurate discriminative performance in PA-type trials than in NA-type trials. This phenomenon has been documented in children, humans with mental retardation (Fletcher et al., 1968) and also in other species such as chimpanzees (Thompson, 1954; Fletcher and Garske, 1972), pigeons (Richards, 1973; Richards and Marcattilio, 1975; Hall, 1980; Urcuioli and Michalek, 2007) and, more recently, starlings (Vasconcelos and Monteiro, 2014).

Two main hypotheses have been proposed to explain the ambiguous-cue problem. On one hand, the *interfering cue hypothesis* (Zeaman and House, 1962; Boyer and Polidora, 1972; Fletcher and Garske, 1972; Berch, 1974) considers only the direct value of the discriminative stimuli that result from reinforcement. Thus, it suggests that the lower accuracy in PA discrimination arises from an approach-approach conflict, caused by both stimuli being reinforced across discriminations. Stimulus A is never reinforced in PA trials, but reinforced in NA trials. In an opposite way, no conflict appears in NA trials because N is never reinforced.

On the other hand, the *value transfer hypothesis* (von Fersen et al., 1991; Zentall and Sherburne, 1994; Zentall and Clement, 2001; Zentall, 2004; Urcuioli and Michalek, 2007) suggests that in PA trials, some of the positive value associated with P is transferred to A through second-order conditioning. Thus, because A will receive additional value in PA trials, it will be strongly preferred in NA trials.

In order to test both hypotheses, Urcuioli and Michalek (2007) manipulated the probability of reinforcement in PA trials using two groups: one exposed to continuous reinforcement and the other exposed to partial reinforcement (50%). Using this experimental design, the two hypotheses make different predictions. The *interfering cue hypothesis* predicts indifference within PA-Partial trials, when the choice of the stimulus P is reinforced 50% of the time. In contrast, the *value transfer hypothesis* predicts lower than 50% choices for stimulus P and, as a consequence, preference for the stimulus A in PA-Partial trials. Urcuioli and Michalek (2007) used this protocol in pigeons (2007). More recently, Vasconcelos and Monteiro (2014) did the same, but they used starlings.

Urcuioli and Michalek (2007) reported that partial reinforcement, as predicted, did not affect the accuracy in NA trials, but affected the accuracy in PA type trials. These results were interpreted by Urcuioli and Michalek as support for the *value transfer hypothesis*.

Recently, Vasconcelos and Monteiro (2014) used almost the same procedure as Urcuioli and Michalek (2007). The few differences were the species that they used (starlings vs. pigeons), the number of trials within each session, and the form of the frontal panel in which the alternatives were presented (**Table 1**). The frontal panel was 40 cm tall with three sections: a middle subpanel 11.5 cm wide, and two side subpanels (equal width) attached to the cage at a 120° angle from the center subpanel. The middle panel had one response key and the food hopper. Each side subpanel had one response key in the center (Vasconcelos and Monteiro, 2014).

**Table 1:** Differences in procedures used by Urcuioli and Michalek (2007); Vasconcelos and Monteiro (2014), and our experiment.

	Urcuioli and Michalek (2007)	Vasconcelos and Monteiro (2014)	Our experiment
Species	Pigeons (*White carneaux*)	Starlings (*Sturnus vulgaris*)	Pigeons (*Columba livia*)
Panel	Flat	120° angle	Flat
Number of sessions/Trials per session	30/60	36/60	18/60
Food deprivation	80%	90%	80%
Type of reinforcer	Food (no more data offered)	Two pellets of food	Five seconds of food access

In their second experiment, Vasconcelos and Monteiro reported that the percentage of correct choices for the group PA-Partial was significantly lower than for the group PA-Continuous across the first sessions (similar to Urcuioli and Michalek, 2007), but after a few sessions, the percentage of correct choices increased and reached high levels of accuracy during the last sessions. Thus, at the end of the experiment, no differences existed when comparing accuracies between PA-Continuous and PA-Partial groups. This high accuracy observed during PA trials is difficult to explain in terms of either of the above-mentioned two hypotheses. Therefore, the ambiguous-cue effect was present but lower than expected. This is in contrast to the results reported by Urcuioli and Michalek (2007).

Vasconcelos and Monteiro (2014) stated that the differences between their results and previous results from Urcuioli and Michalek (2007) could be attributed to differences between species (starlings vs. pigeons) as much as to the different configurations of the frontal panel (120° angle vs. flat). They argued that to achieve such high levels of accuracy within PA trials, the starlings must have attended to configurational cues, thus differentiating stimulus A when presented within PA trials from stimulus A when presented within NA trials. They stated that, perhaps due to the 120° angled frontal panel, the stimuli were perceived simultaneously, triggering configurational perception of the stimuli and precluding the value transfer from occurring. Thus, the stimulus A could be a different stimulus in the presence of stimulus P than in the presence of stimulus N (Vasconcelos and Monteiro, 2014). PA and NA would then operate as two different compound stimuli. In contrast, when the stimuli P, N and A were presented on a flat panel, they must have been sequentially perceived and, as a consequence, they could not have been perceived as a compound. In such a case, stimulus A is the same in both PA and NA trials. Under this condition value transfer is possible via second-order conditioning.

How the stimuli are perceived can account for the differences between Urcuioli and Michalek (2007), using pigeons, and Vasconcelos and Monteiro (2014), using starlings. Whereas the lower initial accuracy within the PA-Partial group reported using both pigeons, and starlings, can be explained by the value transfer hypothesis (von Fersen et al., 1991; Zentall and Sherburne, 1994; Zentall and Clement, 2001; Urcuioli and Michalek, 2007), the progression of accuracy within the PA-Partial group reported by Vasconcelos and Monteiro (2014) using starlings is better explained from an informational value approach. The accuracy within PA trials increases as the starlings learn the informational value of the compound stimuli. During the first sessions, the accuracy is low, but after a few sessions, as the compound stimuli acquire informational value, the accuracy increases. The informational value noted here refers to the certainty with which a particular stimulus anticipates what will immediately happen. As a subject is exposed to a task it attributes predictive value to the stimuli that appear in the context of that task. Therefore, initially the certainty will be low, but with the advancement of the trials, the certainty about what will happen after a stimulus is selected will increase.

With regard to the above-mentioned theories, our aim was to evaluate whether the differences reported by Vasconcelos and Monteiro (2014) as compared with Urcuioli and Michalek (2007) were in fact due to the type of species used or because the angled panel used by Vasconcelos and Monteiro (2014) induced the perception of a compound stimulus. We report the results of one experiment using a procedure similar to the procedure first used by Urcuioli and Michalek (2007) and later by Vasconcelos and Monteiro (2014).

Under this procedure, the two-main ambiguous-cue effect hypotheses make different predictions, particularly regarding the effect of partial reinforcement. This procedure give us two advantages. First, it permits the replication of data reported in Urcuioli and Michalek (2007). Considering that Vasconcelos and Monteiro found different results using starlings, it makes sense to re-test pigeons when studying the ambiguous-cue problem. Second, in the event that we find similar results to those reported in Vasconcelos and Monteiro (2014), even when we might not be able to state that their hypothesis is false — because the stimuli could still be perceived as a compound in spite of a flat panel— at least we could conclude that an angled panel would not be necessary.

The differences between the three experiments are shown in **Table 1**.

## Materials and Methods

### Participants

Twelve pigeons (*Columba livia*) were used. All of these pigeons had previous experience in a peck-response acquisition experiment in an auto-shaping procedure, so they were not pre-trained for the current task. All pigeons were housed individually in 50 × 72 × 40 cm metal cages in a colony room with a 12:12-h day-night cycle with lights on from 07:00 to 19:00. Indoor temperature was constant 25°C. The pigeons were maintained approximately at 80% of their free-feeding weight during the experiment. Water was freely available at all times.

The experimental procedure was approved by the local Ethical Committee of the Center for Studies and Investigations in Behavior, by the University of Guadalajara committee for animal experiments, and met governmental guidelines.

### Apparatus

**FIGURE 1:**
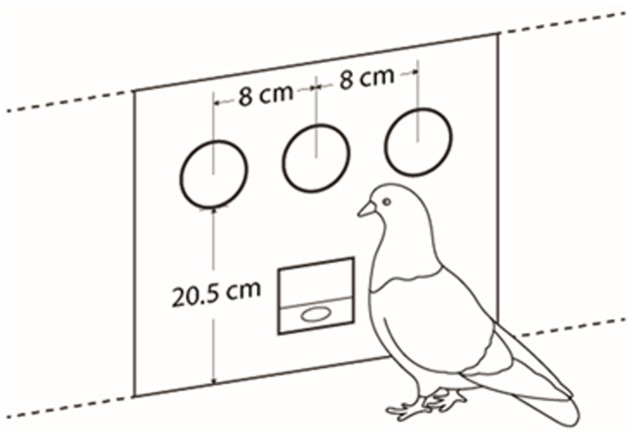
Flat frontal panel and distribution of stimuli and food hopper.

Two operant chambers, acoustically isolated (MED ENV-007, 25.4 cm wide × 21 cm high × 31.8 cm long). The frontal panel of the cages was flat and composed of three subpanels (see **Figure 1**). The middle panel had a food hopper (ENV-123AM). Over the food hopper a 2.5 cm diameter response cue was installed (ENV-123AM), 20.5cm from the floor grid bars. This operated as an attentional flashing cue. On each side subpanel, at the same height as the attentional cue, and separated 8 cm from center to center, one cue (ENV-131M) was installed that operated as a stimulus cue. Each experimental cage was placed inside a sound isolated chamber (ENV-018V) equipped with a fan (VF80A11- AC 115 v). MED-PC IV software was used for programming and recording data.

### Procedure

Pigeons were randomly distributed into two groups: PA-Continuous Group and PA-Partial Group. As the pigeons had previous experience in an auto-shaping task they were not trained to peck the cues and were directly exposed to the experimental procedure.

For all pigeons, a green cue served as the positive stimulus P (S+), a red cue as a negative stimulus N (S-), and a blue cue served as the ambiguous stimulus A. Thus, the blue cue served as a S+ or S- depending on whether it was simultaneously presented with an N or P cue. The attentional cue presented a white flashing light, that turned on and off each 0.5 s, and stayed flashing until it was pecked.

A total number of 18 sessions (one session per day) were run. Each session ended after 60 trials or 4 h from the session start, whichever came first. All trials began with the attentional cue flashing just above the food hopper. A single peck to the center key switched its light off and turned on the stimulus cues located on both subpanels. A single peck to either of these turned them off, and produced either 5 s access to grain or advancement to the next trial, depending on whether the choice was reinforced or not in that specific trial. All trials were consistently separated by a 40 s inter-trial interval (ITI), during which no cues were lighted and the food hopper was disabled.

In half of the trials, after the attentional cue was pecked, the pigeons chose between stimuli P and A (named PA trials). In the other half, after the attentional cue was pecked, the choice was between stimuli N and A (named NA trials), as in Urcuioli and Michalek (2007) and Vasconcelos and Monteiro (2014). On PA trials P was always reinforced for the PA-Continuous Group (PA-CG) but only 50% reinforced for the PA-Partial Group (PA-PG). For both groups, stimulus N was never reinforced, and stimulus A was always reinforced when presented with stimulus N but never reinforced when presented with stimulus P. Stimuli cue allocation was counterbalanced across the trials. The order of the trials was randomized.

## Results

**FIGURE 2:**
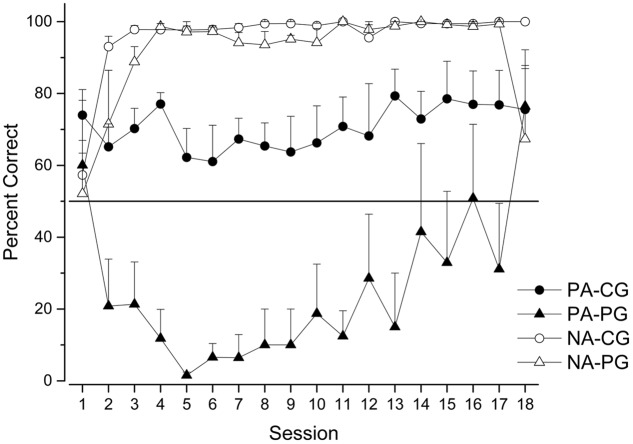
Percentage of correct choices (mean ± SEM) on PA and NA trials for Continuous (CG) and Partial (PG) groups across sessions.

**Figure 2** shows the mean percentage of correct responses on PA and NA trials for both groups session by session.

As in previous studies on pigeons and starlings (Urcuioli and Michalek, 2007; Vasconcelos and Monteiro, 2014) our data show that the performance of pigeons in NA trials is a stable phenomenon. After a few sessions, all pigeons showed very high accuracies. They very frequently chose stimulus A when it was jointly presented with stimulus N for both continuous and partial groups. It is worth noting that one pigeon from the partial group never chose A cue in the NA trials. Therefore, the mean percentage of correct responses decreased in these experiments. Nonetheless, at a descriptive level we observed high stability when compared with previous sessions.

The pattern of results corresponding to PA trials is very similar to the pattern observed across the first 18 sessions of the second experiment using starlings reported by Vasconcelos and Monteiro (2014). We ran only 18 sessions (plotted individually) while Vasconcelos and Monteiro (2014) ran 36 sessions, reported in blocks of 2 sessions. The level of accuracy in the PA-CG trials was greater than random and stable, very close to 75%. More interesting was the pattern of correct responses in the PA-PG sessions. As in Vasconcelos and Monteiro (2014), the accuracy decreased, and after a few sessions increased similar to PA-CG accuracy, crossing the threshold of randomness at the end of the experiment but without reaching statistical significance considering the last four sessions, 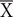 = 47.82, *t*(19) = -0.228, *p* = 0.822.

In sum, our data with pigeons and using a flat panel instead of an angled panel basically reproduced the data previously reported for starlings, but differed from the data reported by Urcuioli and Michalek (2007) for pigeons. In the case of the NA trials, no matter the group, the three experiments report the highest (close to 100%) and a very stable level of accuracy after a few sessions. The pattern observed in the PA-CG trials in the three experiments was stable, but near random in Urcuioli and Michalek (2007) using pigeons and above random in the other two experiments, despite the fact that we used different species and a different panel. Finally, the starlings and our pigeons performed equally in the PA-PG trials, but this performance was different than that observed with the pigeons in Urcuioli and Michalek (2007). While the accuracies in Urcuioli and Michalek (2007) were low and stable after a few sessions, we —and previously Vasconcelos and Monteiro (2014)— report a low level of accuracies across the first sessions followed by an increase to above randomness after 18 sessions, or block number 9 in the Vasconcelos and Monteiro (2014) experiment.

For the analysis, in order to make comparisons between mean percentages of correct choices, only the last four sessions of each group and trial type were considered. One pigeon from the PA-Partial Group was dropped from the analysis because in some sessions no responses were recorded.

**FIGURE 3:**
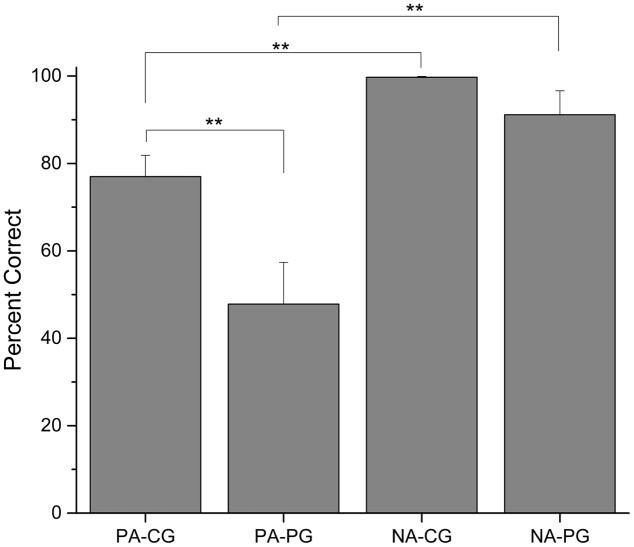
Percentage of correct choices (mean ± SEM) in the last four sessions of each group ^∗∗^*p* < 0.01.

Comparing the last four sessions for each group (see **Figure 3**), there was a statistically significant difference between PA-CG and PA-PG, *t*(42) = 2.854, *p* = 0.007, but not between NA-CG and NA-PG, *t*(42) = 1.714, *p* = 0.094. The difference between PA-CG and NA-CG was also significant, *t*(23) = -4.712, *p* = 0.001, as well as the difference between PA-PG and NA-PG, *t*(19) = -3.475, *p* = 0.003.

## Discussion

The aim of the present experiment was to assess whether the differences between the results of Urcuioli and Michalek (2007) and Vasconcelos and Monteiro (2014) were due to the fact that they used different species, or because the 120° angled panel used in Vasconcelos and Monteiro (2014) induced the perception of compound stimuli, precluding value transfer from occurring.

Our data suggest that neither the species used nor the 120° angled panel adequately explain the different results reported in both studies. Even though we ran 18 sessions, our data reproduced those reported in Vasconcelos and Monteiro (2014) – at least the first nine blocks of two sessions- but using a different species and a different panel. With regard to this, we are assuming that with an extended exposure (more than 18 sessions), PA-PG could have reached an asymptotic accuracy above randomness or even approaching 90%, as observed by Vasconcelos and Monteiro (2014).

The problem is that our data using pigeons differ from those reported in Urcuioli and Michalek (2007), who also used pigeons and a similar flat panel. Therefore, this experiment demonstrates that pigeons can behave in a similar way to starlings in an ambiguous-cue task, and that an angled panel is not necessary to observe a high accuracy in partially reinforced PA trials.

We do not have a clear hypothesis about why our pigeons behaved in a different way than those in the Urcuioli and Michalek (2007) experiment. Our designs were very similar, and we used the same species. Differences in accuracy, particularly within PA trials, could be attributed to fine methodological differences or may be due to individual differences between the samples used. In Urcuioli and Michalek (2007), nine of the twelve pigeons were naïve, while three had previous experience in a two-alternative delay matching experiment. All of our twelve pigeons had previous experience in an autoshaping procedure. This leads to the conclusion that further research should be conducted in order to identify what is controlling the behavior of pigeons in the ambiguous-cue task.

More important to the aim of this study, is the observation that despite the fact that our pigeons basically reproduced the accuracy of the starling and the ambiguous-cue effect experiments, in this case an angled panel was not necessary. To explain this, at least three different possibilities arise. First, it could be feasible that the flat panel does not preclude the perception of cues simultaneously. Thus, some configurational value would be transferred from S+ to S-, despite the fact that the stimuli are not strictly part of a compound. At least they were not simultaneously presented. In this case, the hypothesis proposed in Vasconcelos and Monteiro (2014) would potentially be valid, but further research must be conducted to validate this idea. Second, it is not necessary to perceive the combination of PA and NA stimuli as a compound to preclude value transfer from occurring. In this case, the above hypothesis would be false. Finally, individual differences in discriminative learning could account for the inconsistent data reported in the experiments here reviewed.

Some procedures with pairs of stimuli in which the value transfer theory has been tested, have shown that eventually this theory fails when fitting the data. For example, in transitive inference procedures (see Vasconcelos, 2008, for a review) using flat panels, the subjects are exposed to pairs of stimuli A+ B-, B+ C-, C+ D-, D+E-. Then, when the subjects are tested with the non-adjacent pairs, (AC, BD, CE), in the absence of reinforcement or without differential reinforcement, a preference for B over D is expected as a case of transitive inference, so that the set of pairs becomes a *representation* of A > B > C > D > E. In such procedures, the evidence favoring the value transfer theory remains inconsistent. For example, Lazareva and Wasserman (2006) found that their pigeons could establish a transitive inference even when associative models did not predicted it (including those based on the *value transfer theory*), and even when the associative strength of D was increased. However, in another study (Lazareva et al., 2004) the associative models fit relatively well with the data from transitive inference employing hooded crows (*Corvus cornix)*. Studies with pigeons (Steirn et al., 1995) have also shown evidence supporting the value transfer theory in transitive inference procedures. Regarding the similarity between ambiguous cue procedures and transitive inference procedures, where the correct choice depends on the adjacent pair being trained, it is unclear if the associative processes are sufficient to explain the observed effects.

Additionally, previous research has shown (in monkeys, children and the mentally retarded) that even when performance in NA trials can be better than in PA trials, an improvement in PA performance emerges as a function of prompting, but without partial reinforcement (Fletcher et al., 1968). Thus, the ambiguous-cue task could be interpreted as a case of discriminative learning between compound stimuli, in which accuracy improves across trials (similar to a simultaneous discrimination procedure). Together with previously published work, our data demonstrate that high performances can be observed in both avian species, which supports the idea that the improvement in performance seems to be independent of species differences.

It has been shown (employing a flat touch screen) that a pigeon’s visual discrimination can rely on local and global cues, which partially depends on individual differences. For example, Troje and Aust (2013) found individual differences in the percentage of correct choices where the correct stimulus was a compound with movement features (a set of points representing biological movement: a walking man and a walking pigeon). Based on this finding, they differentiated between pigeons relying on local motion and pigeons relying on global motion. With respect to the same rationale, the effect of selective attention has been shown to affect a pigeon’s performance when the disparity between stimuli (more disparity favors better discrimination) is varied (Teng et al., 2015).

With regard to the above, for ambiguous-cue procedures, a comparison between pigeons with local cue perception and pigeons with global cue perception would be necessary in order to assess value transfer effects, while also considering the possible effect of selective attention as an involved mechanism.

Other procedures have shown that several features of pigeons’ visual perception resemble those in human visual perception. For example, pigeons can discriminate cues varying in deepness and density (Cavoto and Cook, 2006), as well as movement (Troje and Aust, 2013). Along with the above-mentioned, Fujita et al. (1993) replicated the Ponzo illusion in pigeons. Since contextual cues (in this case the parallel lines) are necessary for the Ponzo illusion and since this effect has been found in a flat setting (a TV screen), this finding supports the conclusion that pigeons can respond to contextual cues even with a flat panel. Additionally, there are findings in matching-to-sample procedures in which, despite the employment of a flat panel and compound stimuli, pigeons were shown to be capable of selecting greater than a random percentage of correct choices (Lamb and Riley, 1981; Lamb, 1988), which would be inconsistent with the hypothesis suggested by Vasconcelos and Monteiro (2014). Nevertheless, these findings also suggest that some discriminations become more difficult depending on the way the compound stimuli are configured (e. g., unified, separated or superimposed), an effect that is referred to as the *information-overload hypothesis*. Therefore, due to the fact that the rate of correct responses across sessions can be affected purely by the features of the compound stimuli, by the rate of reinforcement associated with them or by the amount of value transferred between them, the hypothesis about peripheral perception in pigeons (Vasconcelos and Monteiro, 2014), deserves further investigation in the context of pigeons’ visual perception and ambiguous cue procedures.

This kind of inquiry about visual perception could have implications for the value transfer theory and the interfering cue hypothesis.

For example, Enkel et al. (2010) interpreted the ambiguous cue problem in a different way and proposed another protocol. An S+, that operates as an appetitive reinforcer, is associated with a particular value of an auditory discriminative stimuli (i.e., a tone of 300 Hz) whereas an S-, the absence of reinforcer or an aversive stimulus, is associated with another value of the auditory stimulus (i.e., a tone of 500 Hz). During the ambiguous cue test, a third stimulus with an intermediate value (e.g., 400 Hz) is employed in the absence of reinforcement. This procedure can also be explored regarding the results seen in Urcuioli and Michalek (2007), as well as in Vasconcelos and Monteiro (2014), where the ambiguous stimulus was not related to the properties of the stimuli, but instead with the associated outcomes.

Because our data reproduced those reported in Vasconcelos and Monteiro (2014), they cannot be adequately explained by neither the value transfer hypothesis nor the interfering cue hypothesis. Our data contribute to extend the applicability of the data reported in Vasconcelos and Monteiro (2014) to other species and experiments independent of the panel used. Our findings suggest that ambiguous cue learning could be explained as a case of discriminative learning between compound stimuli where performance improves as learning proceeds, regardless of the panel configuration. Additional research on visual perception in pigeons should be conducted in order to advance our comprehension of the ambiguous-cue effect as the specific processes involved during the performance require further attention.

## Author Contributions

The five authors jointly designed the experiment. CE, LÁ, and ZV collected the data. HC-P and ÓG-L jointly wrote the manuscript.

## Conflict of Interest Statement

The authors declare that the research was conducted in the absence of any commercial or financial relationships that could be construed as a potential conflict of interest.
